# A 3’ UTR SNP rs885863, a *cis*-eQTL for the circadian gene *VIPR2* and lincRNA 689, is associated with opioid addiction

**DOI:** 10.1371/journal.pone.0224399

**Published:** 2019-11-05

**Authors:** Orna Levran, Matthew Randesi, John Rotrosen, Jurg Ott, Miriam Adelson, Mary Jeanne Kreek

**Affiliations:** 1 The Laboratory of the Biology of Addictive Diseases, The Rockefeller University, New York, New York, United States of America; 2 NYU School of Medicine, New York, New York, United States of America; 3 The Laboratory of Statistical Genetics, The Rockefeller University, New York, New York, United States of America; 4 Dr. Miriam and Sheldon G. Adelson Clinic for Drug Abuse Treatment and Research, Las Vegas, Nevada, United States of America; Boston University, UNITED STATES

## Abstract

There is a reciprocal relationship between the circadian and the reward systems. Polymorphisms in several circadian rhythm-related (clock) genes were associated with drug addiction. This study aims to search for associations between 895 variants in 39 circadian rhythm-related genes and opioid addiction (OUD). Genotyping was performed with the Smokescreen^®^ array. Ancestry was verified by principal/MDS component analysis and the sample was limited to European Americans (EA) (OUD; n = 435, controls; n = 138). Nominally significant associations (*p* < 0.01) were detected for several variants in genes encoding vasoactive intestinal peptide receptor 2 (*VIPR2*), period circadian regulator *2* (*PER2*), casein kinase 1 epsilon (*CSNK1E*), and activator of transcription and developmental regulator (*AUTS2*), but no signal survived correction for multiple testing. There was intriguing association signal for the untranslated region (3’ UTR) variant rs885863 in *VIPR2*, (*p* = .0065; OR = 0.51; 95% CI 0.31–0.51). The result was corroborated in an independent EA OUD sample (n = 398, *p* = 0.0036; for the combined samples). Notably, this SNP is an expression quantitative trait locus (*cis*-eQTL) for *VIPR2* and a long intergenic non-coding RNA, lincRNA 689, in a tissue-specific manner, based on the Genotype-Tissue Expression (GTEx) project. Vasoactive intestinal peptide (VIP) is an important peptide of light-activated suprachiasmatic nucleus cells. It regulates diverse physiological processes including circadian rhythms, learning and memory, and stress response. This is the first report of an association of a *VIPR2* variant and OUD. Additionally, analysis of combinations of single nucleotide polymorphisms (SNPs) genotypes revealed an association of *PER2* SNP rs80136044, and SNP rs4128839, located 41.6 kb downstream of neuropeptide Y receptor type 1 gene, *NPY1R* (*p* = 3.4 × 10^−6^, OR = 11.4, 95% CI 2.7–48.2). The study provides preliminary insight into the relationship between genetic variants in circadian rhythm genes and long non-coding RNA (lncRNAs) in their vicinity, and opioid addiction.

## Introduction

The circadian clock has a bidirectional relationship with the reward system [1]. Circadian rhythms are physical and behavioral changes that follow a daily cycle and respond primarily to light. Drug addiction is a chronic relapsing disease with a genetic and environmental contribution (e.g., stress), that is characterized by compulsive use and destructive consequences [2]. Internal circadian desynchrony can exacerbate or affect the development of a range of diseases including drug addiction. Exposure to drugs of abuse affects neuronal firing within the suprachiasmatic nucleus (SCN) and produces changes to circadian rhythms that persist even after exposure is stopped and may contribute to return to use [3]. Drugs of abuse modulate the expression of circadian rhythm-related genes in the brain and these genes regulate pathways and neurotransmitter systems that have a role in drug addiction [4–7].

The current case-control association study focuses on selected genes that are related to the circadian rhythm, including core genes that are components of the primary circadian feedback loop and genes that are linked to the core loop or controlled by the circadian rhythm. The circadian clock consists of several proteins that interact in transcriptional and translational feedback loops, including clock, aryl hydrocarbon receptor nuclear translocator likes (ARNTL or BMAL), periods (PER), cryptochromes (CRY), casein kinases (CK), transcription factors like neuronal PAS domain protein 2 (NPAS2) as well as basic helix-loop-helix proteins [8–11]. A central peptide of light-activated SCN cells is the vasoactive intestinal peptide (VIP) that acts through its receptors, vasoactive intestinal peptide receptors 1 and 2 (VIPR1 and VIPR2) [12, 13].

Clock-controlled genes are genes whose transcription is subjected to circadian control by core clock proteins. They include various neuromodulators or neuropeptides. The hypothalamic-pituitary-adrenal (HPA) axis is under circadian regulation and its components display circadian rhythms [14]. The HPA-axis interacts with genetic factors in circadian rhythms genes (gene x environment) to produce addiction risk [6, 15].

Several studies indicated associations of polymorphisms in circadian rhythm-related genes with drug addictions [3, 6]. We have previously reported associations of polymorphisms in some of the genes included in the current study, as these genes play a role in other addiction-related pathways we studied. Associations were identified for *CSNK1E*, *NPY1R*, and *NPY5R* in subjects with European ancestry [16–18]. Although there is overlap between the samples and the genes analyzed in the previous studies and the current study, the current study includes additional polymorphisms in the genes studied previously, as well as additional genes.

The goal of this study was to explore the hypothesis that polymorphisms in genes related to circadian rhythms are associated with susceptibility to opioid addiction. To limit population stratification and increase the power to detect true associations, the study was limited to subjects of European ancestry from the USA, and the case subjects were from the end of the spectrum of opioid addiction.

## Materials and methods

### Discovery sample

The present study includes a subsample of a cohort (n = 1810) that was shared by the Laboratory of the Biology of Addictive Diseases from the Rockefeller University with the National Institute on Drug Abuse (NIDA) Genetics Consortium. The current study is limited to subjects with opioid addiction (OUD) with predominantly European ancestry (EA) from the USA (n = 573). Subjects are assigned to the OUD sample if heroin is their major addiction. They may or may not be addicted to or abuse cocaine or alcohol, as described [19].

Subjects were recruited at specific opiate treatment programs (e.g., Manhattan Campus of VA NY Harbor Health Care System, Weill Medical College of Cornell University, and Dr. Miriam and Sheldon G. Adelson Clinic for Drug Abuse Treatment and Research, in Las Vegas) or at the Rockefeller University.

Ascertainment was made by personal interviews, using the Addiction Severity Index (ASI) [20], KMSK [21] and Diagnostic and Statistical Manual of Mental Disorders, 4th Edition (DSM-IV). All OUD samples had a diagnosis of heroin dependence based on lifetime DSM-IV criteria, had a history of at least one year of multiple daily uses, and were in methadone maintenance treatment at the time of recruitment. Subjects with active DSM-IV axis I disorder were excluded from the study.

The eligibility criterion for the control group was no diagnosis of illicit drug abuse. Subjects with excessive drinking or cannabis use were excluded from the control group.

The study was approved by the institutional review boards of the VA New York Harbor Health Care System and the Rockefeller University (for Rockefeller University and the Las Vegas clinic). All subjects signed informed consent for genetic studies and sharing DNA with NIDA.

### An independent EA OUD sample

An independent EA OUD sample (n = 398) that is not related to the discovery OUD sample, was analyzed for the SNP that gave significant results in the original analysis. This sample is part of a larger sample obtained from the NIDA Clinical Trials Network study (CTN-0051), a comparative effectiveness trial of 24 weeks of treatment of OUD with extended-release naltrexone versus sublingual buprenorphine-naloxone [22]. Subjects were recruited at community treatment programs affiliated with the CTN and were diagnosed with DSM-5 to have OUD. A subsample of this sample (n = 364) was used in our previous study [19] and 34 samples were obtained after the completion of the previous study. All sites obtained local institutional review board approval and all participants signed informed consent for genetic studies.

### SNPs and genotyping

A total of 39 circadian rhythm-related genes were selected *a priori* for the current study (Table 1). The genes were selected based on current literature and include canonical core clock genes as well as circadian rhythm-related genes that may be relevant to drug addiction. A set of 895 high-quality informative variants from the selected genes (±100 kb) were selected from the Smokescreen^®^ array [23] for analysis.

**Table 1:** Selected circadian rhythm-related genes.

Gene	Gene description
*ARNTL*	aryl hydrocarbon receptor nuclear translocator-like
*ARNTL2*	aryl hydrocarbon receptor nuclear translocator-like 2
*AUTS2*	activator of transcription and developmental regulator
*BHLHE40*	Basic Helix-Loop-Helix Family Member E40
*BHLHE41*	Basic Helix-Loop-Helix Family Member E41
*CLOCK*	clock circadian regulator
*CRY1*	cryptochrome circadian regulator 1
*CRY2*	cryptochrome circadian regulator 2
*CSNK1D*	casein kinase 1 delta
*CSNK1E*	casein kinase 1 epsilon
*CSNK2A1*	casein kinase 2 alpha 1
*CSNK2A2*	casein kinase 2 alpha 2
*CSNK2B*	casein kinase 2 beta
*DBP*	D-box binding PAR bZIP transcription factor
*GSK3B*	glycogen synthase kinase 3 beta
*METTL3*	methyltransferase like 3
*MTNR1B*	melatonin receptor 1B
*NPAS2*	neuronal PAS domain protein 2
*NPFF*	neuropeptide FF-amide peptide precursor
*NPFFR1*	neuropeptide FF receptor 1
*NPFFR2*	neuropeptide FF receptor 2
*NPY*	neuropeptide Y
*NPY1R*	neuropeptide Y receptor Y1
*NPY2R*	neuropeptide Y receptor Y2
*NPY5R*	neuropeptide Y receptor Y5
*NR1D1*	nuclear receptor subfamily 1 group D member 1 (REV-ERB alpha)
*PER1*	period circadian regulator 1
*PER2*	period circadian regulator 2
*PER3*	period circadian regulator 3
*PRKAA2*	Protein Kinase AMP-Activated Catalytic Subunit Alpha 2, AMPK
*PRKACA*	Protein Kinase CAMP-Activated Catalytic Subunit Alpha, PKA
*RORB*	RAR related orphan receptor B
*SIRT1*	sirtuin 1
*TEF*	TEF, PAR BZIP Transcription Factor
*TIMELESS*	timeless circadian regulator
*TIPIN*	TIMELESS interacting protein
*VIP*	vasoactive intestinal peptide
*VIPR1*	vasoactive intestinal peptide receptor 1
*VIPR2*	vasoactive intestinal peptide receptor 2

DNA was sent to the NIDA genetic repository at The Rutgers University. The original multi-ancestry sample (n = 1810) was genotyped with the Smokescreen^®^ array at RUCDR Infinite Biologics at The Rutgers University, as part of the NIDA collaborative project of opioid addiction [19]. Smokescreen^®^ is a genome-wide custom genotyping array of biallelic SNPs and simple indels with addiction-related gene content. CEL files of the current study samples were analyzed with Axiom^™^ Analysis Suite 2.0.0.3.5 (Affymetrix, Santa Clara, CA).

Additional genotyping in an independent sample was performed using Taqman^™^ pre-designed assays (C_7556951_1_) (Thermo Fisher Scientific, Waltham, MA, USA) using Applied Biosystems 7900 Real-Time PCR System according to manufacturers’ instructions.

### PC/MDS analysis

Principal/MDS (multidimensional scaling) component analysis was carried out on the original multi-ancestry cohort with a pruned autosomal SNP set of 429,128 autosomal SNPs (r^2^ = 0.8) (Smokescreen^®^ array) [23], using PLINK. The first four MDS components, c_1_ through c_4_, were computed. The two-dimensional graph of c_1_ versus c_3_ showed the clearest separation of data points into distinct groups. Four distinct groups of data points were identified. Focusing on EA, we assumed that each data point represented a bivariate normal distribution, after suitable transformations of c_1_ and c_3_ values. A small number of outliers were identified as those individuals with h_(c1, c3)_ smaller than a suitable constant. All subsequent analyses were carried out for the PC/MDS defined group.

### Statistical analysis

Familial relationships and duplicates were detected via pairwise Identity By Descent (IBD) analyses with the pruned autosomal SNP set of 429,128 autosomal SNPs (r^2^ = 0.8) in PLINK. Duplicates and relatives (PI_HAT > 0.25) were excluded. Pairwise linkage disequilibrium (LD) (D’ and r^2^) was estimated using Haploview 4.2. LD blocks were identified using the D’ confidence interval bound of 0.7–0.98 [24].

Single-SNP association analyses were conducted using PLINK 1.9 [25] by the maximum chi-square test, under dominant or recessive model assumptions. The following filters were used: a. Exact tests for deviation from Hardy-Weinberg equilibrium (HWE) with a threshold of *p* = 0.05/n, where n = total number of SNPs; b. minor allele frequency (MAF) < 0.05; c. missing genotype data (< 94%). A maximum test statistic was also applied to account for the dominant and the recessive model tests, using Sumstat [26]. Correction for multiple testing was performed by permutation test (n = 100,000) for the model that showed nominally significant results, using PLINK. Analysis of one SNP in an independent EA case sample was conducted by logistic regression under the dominant model either separately or by adding the second sample to the original EA case sample.

Conditional analyses were carried out as implemented in Sumstat [27]. For a given SNP (test SNP), a combined association with any other SNP (target SNP) was calculated, one at a time, by dividing the data into three portions according to the three test SNP genotypes and doing a regular genotype association analysis for the target SNP in each of the three portions, suitably combining results from those three portions [27]. Test SNPs were selected based on three tests, (1) the genotype test, (2) an F-test [28], and (3) a maximum test (dominant versus recessive). The four SNPs with the smallest *p*-value (corrected for testing multiple SNPs) were selected as test SNPs. Correction for multiple testing was performed by a permutation test (n = 100,000).

## Results

The discovery sample includes 435 subjects with heroin addiction (cases) and 138 controls of predominantly European ancestry. Ancestry was determined by PC/MDS and was based on a pruned SNP set of 429,128 autosomal SNPs from the Smokescreen^®^ array [23] (see Material and methods).

The current analysis was limited *a priori* to 895 SNPs overlapping 39 circadian rhythm-related genes. From the original SNP set of these genes, 119 SNPs were excluded based on low frequency in the control sample (MAF < 0.05), six variants were removed due to missing genotype data (< 94%), and two SNPs were excluded based on HWE (*p* < 2 x 10^-^)^6^. A set of 64 SNPs were redundant (r^2^ > 0.994). A final set of 704 variants was used for the association analyses.

Comparison of genotype frequency distributions between cases and controls revealed several nominally significant differences but none of them survived permutation analysis. Table 2 lists the SNPs that passed the nominal significance threshold level of *p* < 0.01.

**Table 2:** Top association signals (*p* < 0.01).

SNP	Position (build 38)	Location	Gene	MAF CEU	Test	OUD	Control	*p*	OR	95% CI
rs885863	7:159,028,278	3' UTR variant	*VIPR2*^a^	0.39	R^b^	56/219/159	31/59/48	0.0065	0.51	0.3–0.5
rs3113275	7:70,514,208	intron	*AUTS2*	0.24	R	30/151/249	1/54/80	0.0055	10.0	1.4–74.4
rs11764092^c^	7:70,732,598	intron	*AUTS2*	0.23	D	15/145/273	3/31/104	0.0078	1.79	1.2–2.8
rs135763	22:38,312,399	intron	*CSNK1E*	0.12	D	16/103/316	4/51/82	0.0046	0.56	0.6–0.8
rs1534891^d^	22:38299094	intron	*CSNK1E*	0.13	D	12/96/327	5/45/88	0.0090	0.58	0.4–0.6
rs80136044^e^	2:238,274,081	intron	*PER2*	0.15	D	7/101/327	1/17/120	0.0036	2.20	1.3–3.8
rs3754729	2:238241585	intergenic	*PER2/HES6*^f^	0.31	D	41/202/189	12/47/78	0.0071	1.70	1.2–1.7

^a^ also in *LINC00689*

^b^ referring to the minor allele

^c^ in high LD (r^2^ > 0.8) with SNP rs7805642

^d^ in high LD with SNP rs5750581, moderate LD (r^2^ > 0.4) with rs135763

^e^ in high LD with rs75509863, rs78839410, and rs80136044

^f^ also a variant in lncRNA AC012485.1

MAF, minor allele frequency; CEU, HapMap sample of Northern and Western European ancestry; CI, confidence interval; D, dominant; R, recessive.

### An independent EA OUD sample for replication

*VIPR2* SNP rs885863 was genotyped in an independent EA OUD sample (CTN, n = 398). There was significant difference in genotype frequency distributions of rs885863 between the original control sample (n = 138) and the EA OUD CTN sample (*p* = 0.0054; OR = 0.48; recessive model). When the two EA OUD samples were combined (n = 832), there was a significant difference in genotype frequency distributions of rs885863 between the original control sample and the combined sample (*p* = 0.0036; OR = 0.5; recessive model).

### Conditional analysis of combinations of the two SNPs

Conditional analysis of genotype combinations of the two SNPs revealed an association of *PER2* SNP rs80136044 and SNP rs4128839, located 41.6 kb downstream of *NPY1R*) (*p* = 3.4 × 10^−6^, OR = 11.4) (S1 Table). Specifically, there was a significantly lower proportion of control samples with the combined genotype A/A for rs4128839 (homozygotes for the major allele) and T/T+C/T for rs80136044 (at least one copy of the minor allele) compared to the OUD samples, 0.01 vs. 0.13, respectively) (S1 Table). Notably, the minor T allele of *PER2* rs80136044 was associated with the risk of OUD in the single gene analysis under the dominant model, but there was no significant association of SNP rs4128839 in the single SNP analysis.

### LD and potential functionality

**Fig 1:**
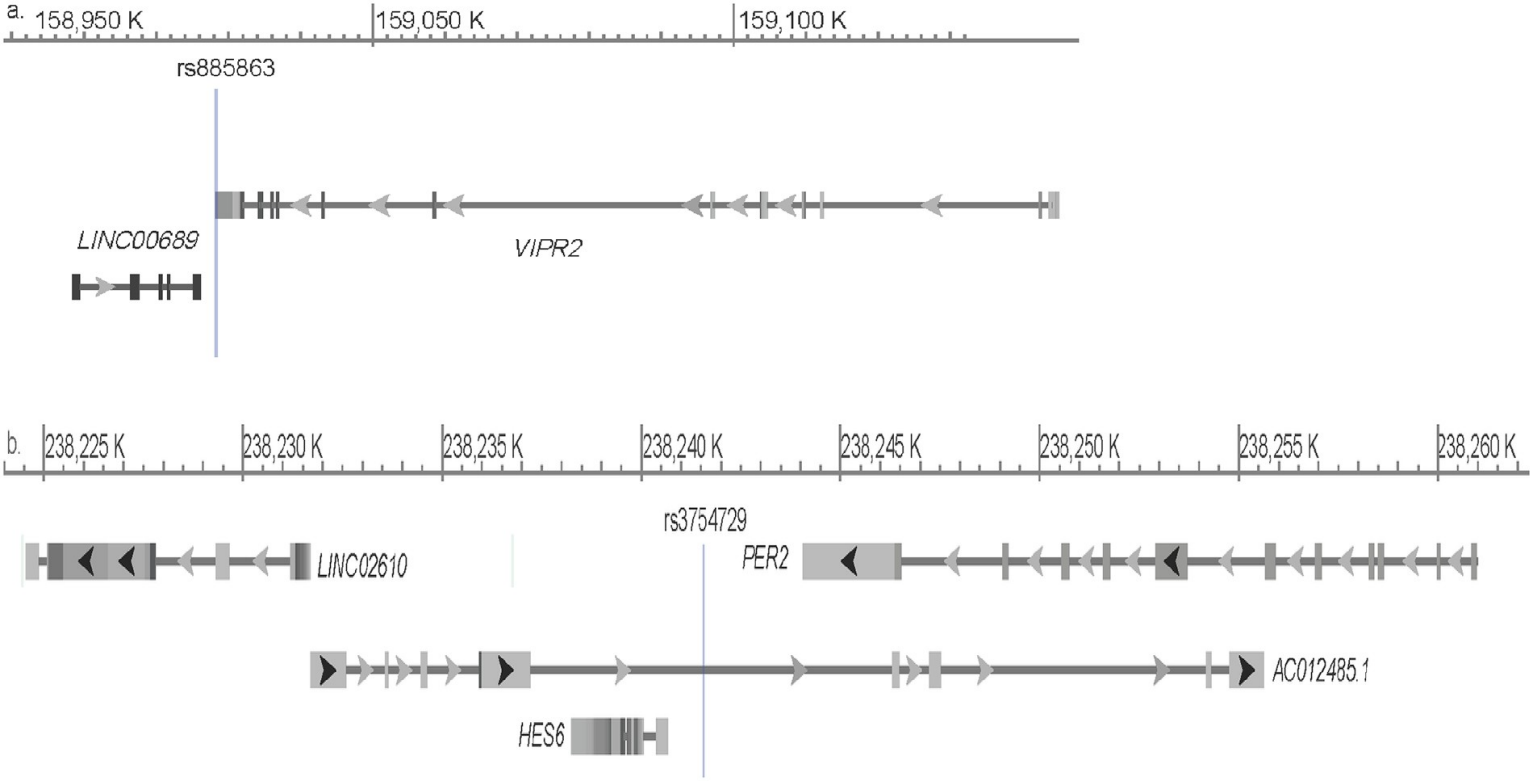
Schematic representation of SNP positions and their eQTL genes. a. *VIPR2* rs885863, b. *PER2*-*HES6* rs3754729.

SNP rs885863 is a 3ꞌ UTR variant in *VIPR2* encoding vasoactive intestinal peptide receptor 2 (also called VPAC2). It is also located in a non-coding exon of long intergenic non-protein coding RNA 689, *LINC00689* (ENSG00000231419) (Fig 1a). SNP rs885863 is in high LD with several 3ꞌ UTR variants as well as intronic variants and non-coding exonic variants in *LINC00689* in CEU (HapMap sample of Northern and Western European ancestry). SNP rs885863 is a *cis*-eQTL for *VIPR2* and an ncRNA-eQTL for *LINC00689*, in a tissue-specific manner (GTEx).

*PER2* rs80136044 is in high LD with three other SNPs included in this study (rs75509863, rs78839410, and rs80136044). It is located in an enhancer region that showed activity in several cell lines. It is in high LD (r^2^ > 0.8) with numerous intronic SNPs in CEU, the synonymous SNP rs2304669 (Ala655 =) as well as SNP rs56386336 in the 3ꞌ UTR. SNP rs2304669 is located in a functional CCCTC-binding factor (CTCF)-binding site and is associated with expression of the Hes family BHLH transcription factor 6 gene (*HES6*), *LINC02610* (ENSG00000186235) and lncRNA AC012485.1 (ENSG00000225057) downstream of *PER2* (GTEx).

SNP rs3754729, indicated in the current study, is localized to the intergenic region between *PER2 and HES6*. It is a regulatory SNP located in a promoter and a CTCF-binding site. It is an eQTL for *HES6*, lncRNAs AC096574.4 (ENSG00000225057.2), and *LINC02610*, in several tissues (GTEx) (Fig 1b). SNP rs4128839 downstream of *NPY1R* is highly conserved and is not in high LD with any SNP in the region.

*AUTS2* SNP rs3113275 is associated with AUTS2 expression in the pituitary (Ensembl). It is also in high LD, in the HapMap CEU, with several intronic variants including the regulatory variant rs3094893 located in an enhancer region.

*CSNK1E* SNPs rs135763 and rs1534891 are in moderate LD (r^2^ = 0.4) in HapMap CEU. The intronic SNP rs1534891 is a regulatory region variant located in a promoter flanking region and a CTCF-binding site.

## Discussion

In the current study, we compared genotype frequencies of polymorphisms in selected circadian rhythm-related genes between subjects with opioid addiction and matched controls, with predominantly European ancestry. Nominally significant associations (*p* < 0.01) were detected for variants in four genes (*VIPR2*, *PER2*, *CSNK1E*, and *AUTS2*), but none survived permutation analysis, possibly due to the relatively small sample size. Nevertheless, specific associations are supported by previous studies, analysis of SNP pair combinations, nominal corroboration in an independent sample and/or evidence for functionality.

### VIPR2

The interesting novel association of the *VIPR2* SNP rs885863 was corroborated in an independent OUD sample of similar ancestry. Notably, SNP rs885863 is also located in a non-coding exon of transcript *LINC00689* and is an eQTL for both *VIPR2* and *LINC00689* in a tissue-specific manner (GTEx). It is in high LD with a second *VIPR2* 3ꞌ UTR SNP, rs885861, that was associated with mood disorders in Spanish subjects [29].

The vasoactive intestinal peptide, VIP, is one of the main peptides of the light-activated suprachiasmatic nucleus that acts through its receptors, vasoactive intestinal peptide receptor 1 and 2 (VIPR1/VPAC1 and VIPR2/VPAC2) [12]. VIP play diverse roles in the central nervous system, including the control of circadian rhythms, learning and memory, psychiatric illness, and responses to stress [30]. *Vipr2* (*Vpac2*) knockout mouse lacks circadian control [30] and also exhibits impaired extinction of cued fear memory and regulation of the dendritic morphology [31]. *VIPR2* is located in the subtelomeric region of chromosome 7 and *VIPR2* duplications were indicated in the etiology of autism [32]. Intriguingly, *LINC00689*, indicated in the current study, was previously associated with obesity in Northern Han Chinese [33], autism [34], and glioma progression [35].

The present study provides further evidence for the role of polymorphisms in *PER2*, *AUTS2*, and *CSNK1E* in drug addiction. The study indicates novel SNPs in these genes and provides information about their potential functionality using bioinformatics tools (e.g., Ensembl and GTEx).

### *PER2*, *HES6*, and *NPY1R*

Intriguingly, an intronic *PER2* SNP, with a nominally significant signal that is in high LD with a synonymous eQTL *PER2* SNP, gave a very strong signal when analyzed in combinations with an intergenic SNP located 41.6 kb downstream of *NPY1R*. There is no functional information about this intergenic SNP.

One of the SNPs indicated, rs3754729, is part of a CTCF-binding site in the intergenic region between *PER2* and *HES6* that includes lncRNA. It is an eQTL for both *HES6* and the lncRNA in several tissues (GTEx). *HES6* encodes Hes family basic helix-loop-helix (bHLH) transcription factor 6 that is controlled by CLOCK [36]. Hepatic Hes6 was upregulated by alcohol feeding in mice [37]. *Hes6* gene has an important role in neurogenesis and neural plasticity.

### AUTS2

Previous reports indicated *AUTS2* functional SNP rs6943555 in association with opioid addiction in Han Chinese [38, 39], and with alcohol consumption in individuals of European ancestry [40]. This SNP was on the array but was not included in the current study because of inadequate cluster separation. It is in moderate LD (r^2^ = 0.60, Dꞌ = 0.87) with SNP rs1880369 that was included in the current study. It is not in high LD with the SNPs indicated to be associated with opioid addiction in the current study.

There is evidence to support the functionality of *AUTS2* SNP rs3113275, indicated in the current study. It is an eQTL for *AUTS2* in the pituitary (GTEx). It is also in high LD, in CEU, with several intronic variants, including SNP rs3094893, a regulatory variant in an enhancer region that was associated with intelligence [41]. Interestingly, AUTS2 is suggested to contribute to the evolution of human cognitive traits [42]. *Auts2* gene expression is increased by repeated cocaine administration in rodents and Auts2 is a target for cocaine-induced chromatin modifications [43].

### CSNK1E

The study supports our previous report of *CSNK1E* SNPs (rs135763 and rs1534891) [16, 17]. *CSNK1E* SNPs were also associated with opioid addiction in Han Chinese [44]. The lambda and epsilon casein kinase 1 isoforms are involved in post-translational regulation of the circadian rhythm, but they are also involved in reward, learning and memory, and cellular growth [45].

### lncRNAs

One of the novel findings of the study is that several of the associated SNPs are eQTL for long noncoding RNAs (lncRNAs) located in the studied gene’s regions. LncRNAs are transcripts with > 200 nucleotides that are not translated into proteins. Long intergenic noncoding RNAs (lincRNAs) are lncRNA which do not overlap protein-coding genes. LncRNAs are important regulators in human disease and have been associated with drug addiction [46]. LncRNAs are involved in gene expression, chromatin remodeling, RNA stabilization and transcription regulation with tissue specificity but their contribution to the development of drug addiction is largely unknown [47, 48]. Specific lncRNAs were shown to be more abundant in post-mortem nucleus accumbens of people who use heroin compared to matched controls [49, 50]. We have recently reported an association of a non-coding *CRHR2* SNP, a *cis*-eQTL for a downstream lncRNA AC005154.6 with opioid addiction [19]. Our study also supports previous studies indicating that a variant may affect several target genes, which may share a regulatory mechanism [51].

In addition to their role in the circadian rhythm, the genes indicated in the study play diverse roles in other addiction-related pathways including reward, learning and memory, anxiety-related behaviors, and response to stress. Therefore, the associations indicated in this study may not necessarily be directly related to the circadian rhythm.

In conclusion, our data support the role of several polymorphisms in circadian genes and lncRNAs in their vicinity, in the susceptibility to opioid addiction. It implicates *VIPR2*, *LINC00689*, and *HES6* for the first time. Further studies are warranted to confirm these preliminary findings.

## Supporting information

S1 Table Conditional analysis of two SNPs genotype combinations. (DOCX)
