# Supplementary material for: A 3’ UTR SNP rs885863, a cis-eQTL for the circadian gene VIPR2 and lincRNA 689, is associated with opioid addiction
Source: PLoS One. 2019 Nov 5;14(11):e0224399. doi: 10.1371/journal.pone.0224399 (PMC6830932; doi:10.1371/journal.pone.0224399)
Supplement: S1 Table — (DOCX) [file pone.0224399.s001.docx]

**S1 Table. Conditional analysis of two SNPs genotype combinations**

|  | **Control** | | | |  |  |  | **OUD** | | | |  |  |
| --- | --- | --- | --- | --- | --- | --- | --- | --- | --- | --- | --- | --- | --- |
|  |  | rs4128839 | | |  |  |  |  | rs4128839 | | |  |  |
|  |  | GG | AG | AA | n | f |  |  | GG | AG | AA | n | f |
|  | TT | 1 | 0 | 0 | 1 | 0.01 |  | TT | 0 | 3 | 4 | 7 | 0.02 |
| rs80136044 | CT | 1 | 14 | 2 | 17 | 0.12 |  | CT | 13 | 33 | 53 | 99 | 0.23 |
|  | CC | 11 | 51 | 58 | 120 | 0.87 |  | CC | 30 | 152 | 145 | 327 | 0.75 |
|  | n | 13 | 65 | 60 | 138 |  |  |  | 43 | 188 | 202 | 433 |  |
|  | f | 0.09 | 0.47 | 0.43 |  |  |  |  | 0.1 | 0.43 | 0.46 |  |  |

*p =* 3.4 × 10^-6^, OR = 11.4
